# Cross-Sectional Survey of Public Perception of Commercial Greyhound Racing in New Zealand

**DOI:** 10.3390/ani14020207

**Published:** 2024-01-08

**Authors:** Kristie E. Cameron, Alison Vaughan, Marie J. McAninch, Kayla Briden, Arnja Dale

**Affiliations:** 1 Unitec, Auckland 1025, New Zealand; 2 Royal New Zealand Society for the Prevention of Cruelty to Animals, Auckland 0640, New Zealand

**Keywords:** greyhound racing, animal welfare, social license, survey

## Abstract

**Simple Summary:**

Commercial greyhound racing in New Zealand has been under scrutiny for its animal welfare practices for over a decade. In 2021, the greyhound racing industry was placed ‘on-notice’ by the New Zealand Racing Minister and Associate Minister of Agriculture (Animal Welfare) due to ongoing issues with data recording, transparency, and animal welfare generally. Social license was identified as a key factor in deciding the future of commercial greyhound racing in New Zealand. This paper describes the findings of a robust survey of the New Zealand public’s opinion towards commercial greyhound racing. The majority of survey respondents reported a negative view of the greyhound racing industry and indicated that, given the chance, they would vote in support of a ban on greyhound racing. The respondents reported a lack of involvement and knowledge of industry practices. These results confirm that the majority of the New Zealand public do not currently support the continuation of commercial greyhound racing in New Zealand.

**Abstract:**

The commercial greyhound racing industry in New Zealand is struggling with an eroding social license and ‘on-notice’ status. Multiple independent reviews of the industry have identified ongoing issues of animal welfare during and between races, euthanasia decisions, poor data tracking, a lack of transparency and problems with rehoming dogs, resulting in New Zealand animal advocacy agencies and the general public questioning the continuation of greyhound racing. The current paper assessed the New Zealand public’s awareness and familiarity with commercial greyhound racing, identified current levels of public support or opposition for racing, and provided context in terms of engagement with greyhound racing using a comprehensive survey of a robust sample of New Zealanders. The results confirm that the social license of the greyhound industry is under challenge with most respondents expressing disagreement with or lack of knowledge of current industry practices and indicating they would vote in support of a ban. There is scope for increasing public acceptability by addressing welfare issues, increasing awareness of positive industry practices, and encouraging transparency of the greyhound racing agency. However, as greyhound racing is on the decline worldwide, calls are likely to continue for a phase-out of commercial greyhound racing.

## 1. Introduction

Commercial greyhound racing has been part of New Zealand’s gambling industry since 1934 as an evolved version of ‘coursing’ [1]. Greyhound racing originated from hare coursing, a sport of European and British nobility in which prize-winning greyhounds (*Canis lupus familiaris*) would hunt a live hare [2]. In 1926, the hunt was replaced by racing on an oval track [3], and in 1954, the live lure was replaced with a synthetic ‘drag hare’ which is kept just out of reach of the greyhounds racing to catch it. Nowadays, greyhound racing leverages the breed’s speed, size, and prey drive to maintain an activity for gambling. Globally, commercial greyhound racing currently operates in six countries: Australia, Ireland, Mexico, New Zealand, the United Kingdom, and the United States of America. The only existing track in Vietnam closed in March 2023 [4]. While people involved in greyhound racing may benefit from this industry, the industry is shrinking and there is public pressure to ban commercial greyhound racing in countries where it currently continues (e.g., Australia [5,6], United Kingdom [7,8,9,10,11,12], and USA [13,14,15]).

The decline in greyhound racing globally is largely driven by an eroding ‘social license’ due to high profile welfare issues and changing public attitudes towards animal welfare and the use of animals in entertainment. A social license is the implicit means by which a community approves or accepts an industry and indirectly its practices. Social licenses exist on a spectrum where the more trust an industry has, the stronger their social license; a social license may be withdrawn, accepted, or approved depending on the level of trust that industry instils in stakeholders [16]. Subsequently, public perception can affect what changes a governing body chooses to implement in terms of rules and regulations [17]. The social license of an industry or activity can be observed through public opinions (for example, surveys) or in public action (for example, attending an event or boycotting it).

Greyhound Racing New Zealand (GRNZ) (Tradename of the New Zealand Greyhound Racing Association Incorporated (NZGRA) and is an Incorporated Society consisting of ten registered member Greyhound Racing Clubs throughout New Zealand. GRNZ is formally recognized in the Racing Industry Act 2020 as a constituent part of the New Zealand Racing Industry) [18] is the governing body for greyhound racing in New Zealand and represents a profitable industry contributing $92.6 million per year to the New Zealand economy, although this is just 5% of the overall racing value in New Zealand according to the New Zealand Racing Board, 2018 (New Zealand Racing Board known as TAB New Zealand and governed by a board of seven members appointed by the Minister for Racing) [19]. The industry has seen comparatively small financial success in comparison to horse racing in New Zealand and has been dogged with scandals and issues with data recording, the transparency of activities, and animal welfare generally [16,17,18,19,20].

An increase in public pressure and advocacy by organizations has prompted multiple reviews of the industry [16,17,18], petitions calling for an end to greyhound racing in New Zealand [21,22,23] and driven government intervention. Since concern was first raised in 2012, numerous independent reviews have resulted in attempts to encourage best-practices, governance, standards, breeding, information tracking, and industry awareness of animal welfare.

A 2021 review concluded that the social license of the greyhound racing industry in New Zealand was at risk due to lack of transparency, poor data recording, and ongoing animal welfare issues [16]. Further, a 2022 report from the Petitions Select Committee stated “We have doubts about whether the greyhound racing industry still has a social license to operate in its current form [24]. It is increasingly clear that the social license is key to the future of greyhound racing. The previous Minister for Racing, Hon Grant Robertson, placed the industry formally “on notice”, stating, “This is a very clear signal, I believe the clearest signal that’s been given to this industry… it is either things improve, or they lose their social license to exist” [25,26].

Polls in some of the countries where commercial greyhound racing remains legal have found strong support for a ban, including an ongoing poll in the New Zealand Herald where currently 70% of 6000 voters are in support of banning greyhound racing [27,28,29,30,31,32,33]. While the observable consensus from these outlets is overwhelmingly supportive of ending commercial greyhound racing, in New Zealand, the views of the industry that are observable are typically individual opinions, found in opinion pieces for media sources [34,35], or non-scientific polls run by media outlets [28,29,30]. Additionally, greyhound racing provides community social benefits and thus there may be areas that support the continuation of the industry [19].

There is currently no large-scale, evidence-based, systematic research examining the New Zealand public’s opinion regarding greyhound racing. The current paper aims to evaluate the social license of commercial greyhound racing in New Zealand. In order to do this, we aim to measure the current awareness of and familiarity with commercial greyhound racing, understand current attitudes and perceptions of the industry, identify the level of current support or opposition for greyhound racing, and provide context in terms of current, relevant behaviors of the public in relation to greyhound racing using a comprehensive survey of a robust sample of New Zealanders. Based on the results of previous surveys in the United Kingdom and Australia, it is expected that public opinion will lean towards negative perceptions of the greyhound industry.

## 2. Materials and Methods

The survey was commissioned by the RNZSPCA. To ensure objectivity, the questionnaire was designed and conducted by Insights HQ Limited, an independent NZ market research agency. The authors employed by the RSPCA (AV, MJM & AD) provided scope and information including the Hansen and Robertson reports as a basis for construction. Further, authors external to the RSPCA (KC & KB) analyzed the data and wrote the core paper; with the authors from the RSPCA providing editorial input. Thus, the integrity of the research procedure and conclusions were maintained.

The questionnaire was fielded with my2cents panelists. The my2cents research community is a cross-section of New Zealanders who have agreed to participate in market research surveys, discussions and focus groups, and is currently used by several major NZ brands and government agencies to conduct representative and robust market research. Further, all my2cents panelists had been pre-profiled so Insights HQ can target specific demographic quotas to ensure data is representative of the New Zealand population, with the ability to verify data, thereby driving accuracy and confidence.

To ensure a nationally representative survey sample of the population, a multi-cell quota design was used to identify even distribution across age, gender and region. For example, instead of surveying 50% male or female, which can result in an uneven number of respondents (e.g., all males from one place), the interlocked cell design uses quotas based on age, gender, and region from the NZ Census data. This ensures that the sample has a similar proportion of respondents that fit each of 50 combinations of age, gender, and location (See Supplementary Data). This data shows the quota that was required for each age, gender, region subsegment and the proportion of the target sample obtained.

Prior to conducting the survey, a technical pilot launch had been conducted. 100 people (that had agreed previously to do market research) were invited to respond and 65 completed the pilot. The purpose of the pilot launch was to check if the survey functioned on different devices and to ensure clarity in how questions were written.

A random sample of 2124 respondents proportioned from the multi-cell design, were invited to participate. Each was invited by email to complete an online questionnaire between 21 and 28 September 2022. A total of 1327 interviews were completed, with 70 respondents starting but not completing the survey. Incompletes were excluded from analysis. Survey questions focused on familiarity with the greyhound racing industry and gambling, governance, agreement with statements regarding welfare, and recommendations sourced from the Robertson [16] report (See Supplementary Data).

The data was analyzed in Excel (Microsoft Corp, Redmond, WA, USA). Descriptive data in the form of percentages of respondents providing a particular response are presented.

## 3. Results

### 3.1. Demographic Information

There were 1327 usable responses to the survey (Table 1). Most identified as NZ European, with Māori and Pacific ethnicities making up 10.3% of respondents. The largest age groups were 65+ years, 25–34 years, and 35–44 years, respectively. Most respondents lived in an urban center (1099/1327, 82.8%).

### 3.2. Engagement with Commercial Greyhound Racing

In terms of engagement with the industry, 50.4% of respondents (669/1327) reported no previous engagement with greyhound racing, either in support or opposition (Table 2). A fifth of respondents reported they had watched a race on television (304/1327, 22.9%), listened or read about commercial racing (305/1327, 23.0%). Only 3.2% of respondents reported they had bet on a greyhound race in the last year (40/1327) compared to 11.5% betting on horse racing (153/1327) and 80.3% not betting on any sports (1065/1327). Most respondents considered it very unlikely they would attend (1141/1327, 86.0%) or bet on (1165/1327, 87.8%) a greyhound race in the next six months, in comparison with those respondents indicating they would be very likely to attend or bet on a greyhound race (36/1327, 2.7%; 29/1327, 2.2%, respectively).

### 3.3. Knowledge of Commercial Greyhound Racing

Respondents were asked about their knowledge of commercial greyhound racing in New Zealand (Table 3). Most respondents had at least heard of or had some level of knowledge about the industry (1160/1327, 87.4%) and less than half were aware of the industry association body, Greyhound Racing New Zealand (533/1327, 40.2%). 42.1% (187/444) of those who considered themselves to have ‘a little bit’ or ‘a lot’ of knowledge about commercial greyhound racing had previously signed a petition against commercial greyhound racing. Most reported they were unaware that the greyhound racing industry had been placed ‘on notice’ for shutdown if greyhound welfare, transparency, and data recording are not addressed (1071/1327, 80.7%).

### 3.4. View of Commercial Greyhound Racing

**Figure 1:**
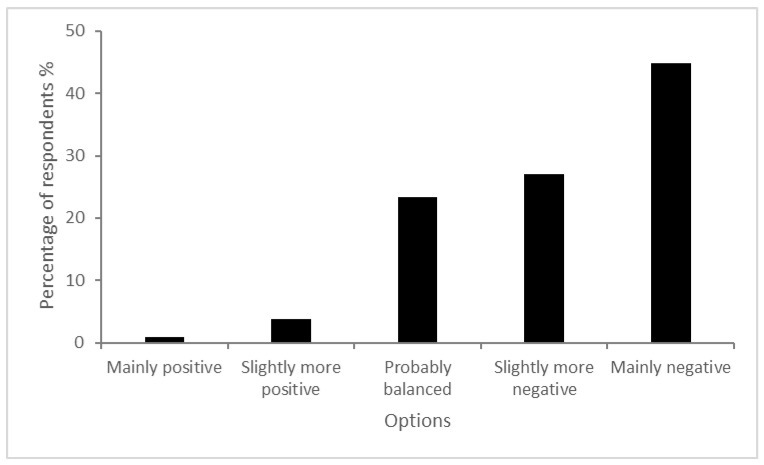
Question: Some people think there are positives to commercial greyhound racing while others think there are negatives. On balance, do you think there are more positive or negative aspects? (*n* = 1327).

The majority of respondents held a negative view of greyhound racing, with 72% having reported either a slightly more negative or mainly negative view of the industry (Figure 1). In contrast, just 4.7% of respondents reported a slightly more or mainly positive view of the industry.

**Figure 2:**
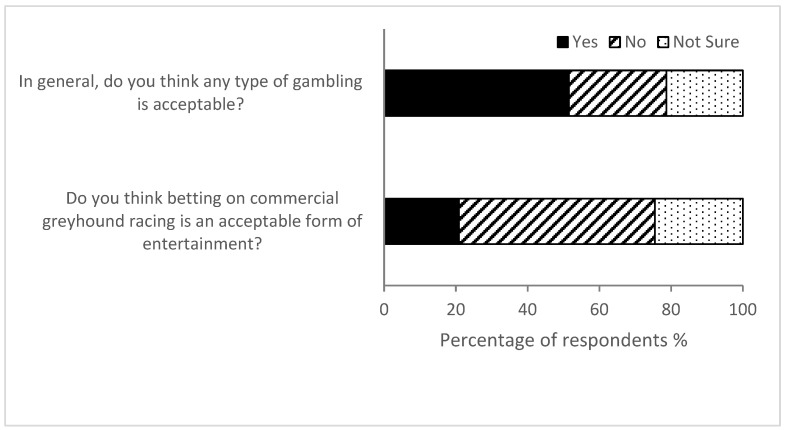
Responses to questions on acceptability of gambling on greyhound racing and gambling in general (*n* = 1327).

While 51.1% of respondents reported they consider gambling in general is acceptable (683/1327), 54.6% believed gambling on greyhound racing was unacceptable as a form of entertainment (725/1327) (Figure 2).

Respondents were asked about their opinion on the future of commercial greyhound racing in New Zealand (Table 4). Of the respondents, the majority, 60.4%, would support a ban (801/1327), 8.2% would not (110/1327), and 31.3% (416/1327) need more information. However, if the call for a referendum was held, 74.8% (992/1327) would vote in support of a ban on greyhound racing. Furthermore, 58.8% of respondents stated they would be disappointed in the government if they did not ban racing (780/1327), 68.3% (906/1327) agree that a ban on the greyhound racing is the right thing to do, and 65.5% (869/1327) believe it would improve New Zealand’s reputation for animal welfare.

Respondents were asked how much they supported the continuation of greyhound racing. Most respondents opposed the continuation; 32.3% strongly opposed (428/1327) and 24.7% somewhat opposed (328/1327). While 35.9% of respondents were indifferent (476/1327), 5.8% somewhat support (77/1327), and 1.3% strongly support the continuation of racing (17/1327); 79.9% of these supporters were NZ European (74/93) and 72.4% identified as male (67/93). Further, of the respondents, 49.5% felt it unacceptable for brands to support commercial greyhound racing (657/1327) with 58.5% (776/1327) feeling more negative about brands that supported the industry. Approximately a third of respondents did not have an opinion about the acceptability of brand support (451/1327, 34.0%) or were unsure about their feelings towards those brands (495/1327, 37.3%).

### 3.5. Positive and Negative Aspects of Commercial Greyhound Racing

**Figure 3:**
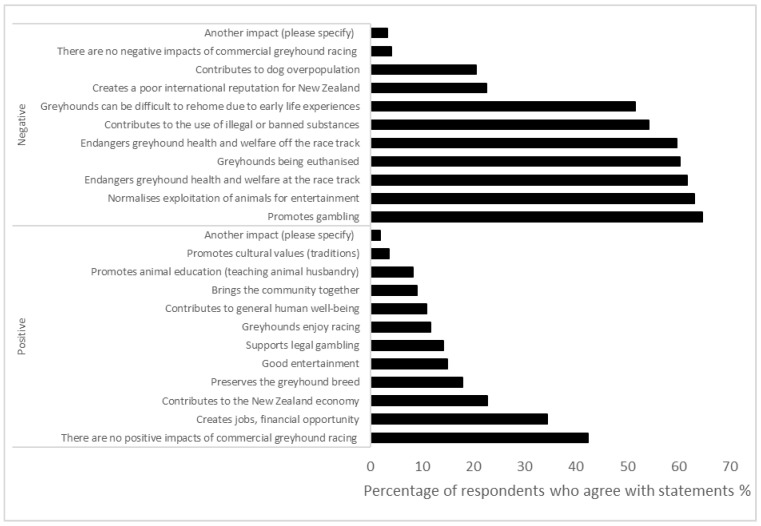
Respondents’ agreement with statements about positive and negative statements aspects of the industry (*n* = 1327).

Respondents showed higher levels of agreement with statements related to negative aspects of commercial greyhound racing than positive aspects (Figure 3). Of the respondents, 42.3% (561/1327) considered that there are no positive impacts of the greyhound racing industry. 34.4% indicated that the industry created jobs and financial opportunity (456/1327), 22.8% believed the industry contributed to New Zealand (302/1327), and 18.0% thought the industry preserves the greyhound breed (239/1327). Contrary to this, more than 60% of respondents answered that the industry endangered greyhound welfare on (817/1327, 61.6%) and off (791/1327, 59.6%) the racetrack, resulted in euthanizing greyhounds (799/1327, 60.2), promoted gambling (857/1327, 64.6%), and normalized exploitation of animals for entertainment (837/1327, 63.1%). Of the respondents, 9.3% (123/1327) reported that they believed greyhounds that are bred for racing have a good life and 47.3% (628/1327) were not sure, while the remainder believed that greyhounds do not have a good life (576/1327, 43.4%).

**Figure 4:**
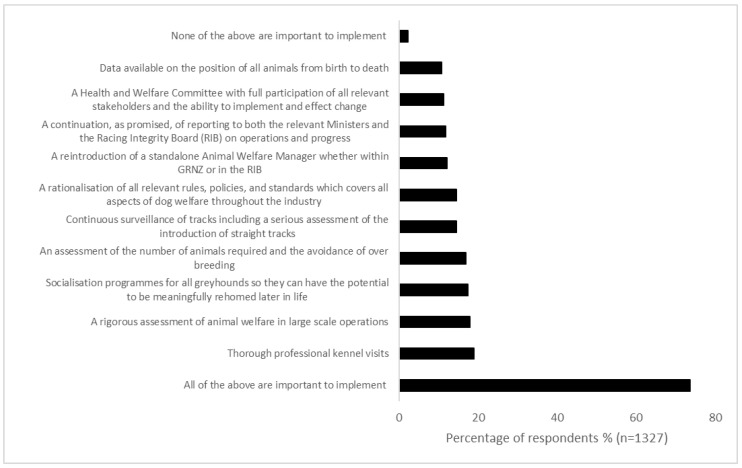
Percentage of respondents and level of importance for the implementation of recommendations in the Robertson Report [16].

Respondents were asked which of the recommendations from a list sourced from the Robertson [16] report were important to implement (Figure 4). Most respondents reported that all should be implemented (970/1327, 73.1%) with thorough professional kennel visits (to check for compliance with welfare standards; 252/1327, 19.0%), rigorous assessment of animal welfare in large scale operations (239/1327, 18.0%), and introduction of socialization programs for all greyhounds (231/1327, 17.4%) the most important recommendations for improving greyhound welfare.

## 4. Discussion

The purpose of this research was to investigate the public perception of greyhound racing and to measure the level of social license greyhound racing currently holds in New Zealand. This is of particular importance as social license has been identified as key in informing the future of the industry and how organizations proceed in the face of the greyhound racing being ‘on notice’ and at risk of closure. The take home message from the results of the survey is that, out of 1327 responses from a robust and representative sample of New Zealanders, most hold a negative view of commercial greyhound racing. Most do not consider betting on greyhounds acceptable, do not believe greyhounds ‘have a good life’, would vote for banning commercial greyhound racing in a referendum, and support a ban of commercial greyhound racing.

Welfare issues within greyhound racing have resulted in a negative perception of the industry; 72% of respondents in this survey reported holding a negative view of commercial greyhound racing. This was similar to the results of surveys of Australians [33] with more than 60% of respondents agreeing that the greyhound industry endangers greyhound welfare on and off the racetrack, results in euthanizing greyhounds, promotes gambling and normalizes exploitation of animals for entertainment.

Responses to questions of the current awareness and familiarity with commercial greyhound racing indicate that half of respondents know of commercial greyhound racing but do not know the details of the industry, have not engaged with greyhound racing in any form, do not intend to do so, and believe that greyhound racing is unacceptable. While many respondents did not report detailed knowledge of the industry, social license is based on public opinion of topics where they may have varying levels of knowledge. This may offer opportunities for both supporters and opponents of the industry to influence public opinion.

Based on the survey results, gambling on greyhound racing was considered less acceptable than gambling generally. Half of respondents indicated that gambling in general was acceptable in contrast to a fifth who considered gambling on greyhound racing acceptable. Approximately double the number of respondents considered gambling on greyhound racing unacceptable in comparison to gambling generally (55% vs. 27%). A minority (14%) responded that greyhound racing supporting legal modes of gambling is a positive attribute of the industry. Only 4% of respondents, that is 53 people, indicated they were likely to engage with greyhound racing in the next 6 months. Our results indicate that gambling concerns alone could not explain respondents’ negative view of commercial greyhound racing. Studies examining attitudes to betting on horse racing found that both concerns about gambling and about animal welfare can influence opinions and stated behaviors related to betting on animal races [36].

The majority of survey respondents appear to oppose greyhound racing in comparison to the small number of people that actively support greyhound racing. Supporters of greyhound racing are typically those who are involved in or engage with the industry. This may indicate an eroding social license for greyhound racing by the majority of the New Zealand public. However, efforts by small numbers of racing advocators, in collaboration with gambling lobbyists, the general racing community, and government actors, were able to overturn the temporary ban of greyhound racing in New South Wales in 2015 [5]. This indicates that it is not only the power of public opinion that informs the decision to continue greyhound racing but those with influence.

Further, there is a tendency of animal use industries to respond to social license pressure by employing public relations strategies. GRNZ embarked on a public relations campaign in early 2022 including filming videos and establishing a website of positive stories, but it is unknown if the positive stories showcased on this website had any impact on public opinion [37].

The lack of science-based research surrounding the public perception of greyhound racing may have contributed to governing bodies’ lack of awareness of risk to the industry’s social license. Polls conducted by media outlets give a snapshot of the public’s perception (e.g., those conducted by TFN in Scotland and Lateline in Australia) [27,29]. These viewpoints, however, are restricted to consumers and users of the media outlet, cannot be considered a representative sample of the whole population, and lack the robustness of surveys that represent the population, such as the current paper. It is difficult to justify a policy change from the results of such polls. The current study provides additional, credible evidence of the industry’s eroding social license in New Zealand to inform decisions about the future of greyhound racing in this country.

A vigorous UK-based survey that found little support or interest in greyhound racing [32] added weight to a campaign challenging the social license of greyhound racing in the UK. The social license of greyhound racing is under public pressure in the UK after a campaign called “Cut the Chase” by the Blue Cross, Dog’s Trust, and RSPCA UK [38]. This has led to discussion of a ban in the Senedd Cymru (Welsh parliament), English and Scottish parliaments [7,9,10]. However, as shown in countries such as Australia, a failing social license does not always result in policy change [5].

Although there is seemingly overwhelming favor for banning greyhound racing in most countries where it is legal, results from polls in Ireland provide conflicting results when measuring public opinion. In a 2019 poll conducted by an independent research group RED C, 66% of respondents thought funding to the greyhound racing industry should be cut [31]. Whereas a follow-up poll conducted by The Journal (a news media outlet) in 2020 found that 72% of respondents would support continued funding to the industry [30].

More robust and comprehensive research has been conducted globally by independent research companies. In 2022, a YouGov survey in the UK found that 91% of respondents do not follow or participate in greyhound racing and 64% regard greyhound racing as unimportant to British culture [32]. In addition, a survey conducted by Lonergan Research, commissioned by the Green Party in Australia, found that 54% of respondents endorse a ban of greyhound racing and 55% believe it is cruel to use dogs and horses for entertainment and gambling [33].

The public’s awareness of issues in an industry will affect that industry’s social license. For example, in 2015, an Australian broadcasting program exposed the animal welfare issues within the greyhound racing industry, thus increasing the awareness of the Australian public [39]. This awareness contributed to the deterioration of the greyhound racing’s social license and prompted multiple petitions to be formed to ban greyhound racing across Australia [39].

Current knowledge about greyhound racing in New Zealand is limited. 70% of respondents were not aware of Greyhound Racing New Zealand as the governing body for racing and 54% did not know the state of greyhound racing globally or that New Zealand has two Ministers that oversee the industry, the Minister for Racing and Associate Minister of Agriculture (Animal Welfare). However, this lack of knowledge provides scope for agencies supporting a ban to provide more context to the public about animal welfare concerns related to the industry, and for GRNZ to improve the welfare of dogs in their industry, data recording, and the transparency of practices within greyhound racing, rather than reacting to scrutiny with closed doors or attempts to change public perception solely through public relations campaigns [15]. Interestingly, increased awareness of an industry’s practices may not produce the intended results and can fail to engender acceptance of that industry, challenging the knowledge deficit model [40].

Steps taken by GRNZ in response to criticism may also reduce trust in the industry. For example, in response to a petition calling for a ban on commercial greyhound racing, the Petitions Select Committee was critical of the removal of the RNZSPCA representative on the GRNZ Health and Welfare Committee stating “In our view, the removal of the RNZSPCA from the health and welfare committee shows poor judgement of what is needed for the industry to keep its license to operate. Shutting out an organization that, while it opposes GRNZ’s work, is prepared to help it improve its practices, has worked against the industry” [24] (page 18).

If the industry was to continue, its social license would need reparation through improvements to welfare, data recording, and transparency. All of the Robertson Report recommendations were considered “important to implement” by over 73% of respondents. These recommendations include avoidance of over breeding to reduce pressure on rehoming, compliance auditing of kennels, socialization programs to prepare greyhounds for life after racing, rationalization of rules and policies for greyhound welfare, a health and welfare committee with full participation of all relevant stakeholders, and data transparency. However, these recommendations were also requirements of previous reviews of the greyhound racing industry [16,17] and failure to address the recommendations of previous reports resulted in the current ‘on notice’ order from the Racing Minister.

In response to a petition by the Greyhound Protection League New Zealand, the government Petitions Select Committee made up of MPs from across the political spectrum responded in a report in 2022 that “We have doubts about whether the greyhound racing industry still has a social license to operate in its current form” [24]. However, at this time the Racing Minister as not made a decision to the future of greyhound racing in New Zealand and the future of the industry remains unclear.

The data was sourced from a sample that was representative of the national population. This means that the sample is proportional to the ratio of New Zealanders within each variable by age, gender, and region. However, greyhound racing is more popular in regional areas because it provides users with an opportunity for social connection, recreational activity and to benefit financially [16,39]. In these areas, racing is a part of community events, charities, and clubs which are made up of volunteers, sponsors, commercial partners, trainers, owners, and the public [19].

Further, the greatest customer expenditure on racing is from the Hawkes Bay region which accounts for 51.7% of customer spending, while the most urban center (Auckland) makes up 37.8% of racing customer expenditure [19]. It is important to consider that the population of racing supporters is likely skewed to the rural regions and, although the sample in this survey was robust in the proportion of respondents from each location, there is greater support for greyhound racing in the rural sector [19]. Specific enquiry into regional participation in greyhound racing might be required to appreciate the effect these communities have on the social license, and thus continuing commercial greyhound racing.

## 5. Conclusions

Commercial greyhound racing in New Zealand has been under scrutiny for its animal welfare practices for over a decade. The results from this study indicate that most people of a robust sample of New Zealanders, given the chance, would vote for a ban on commercial greyhound racing. Therefore, we consider that the acceptance of greyhound racing has declined due to concerns about poor welfare outcomes for dogs and the industry has lost its social license. There is scope for increasing the social license of the general public by improving animal welfare, data recording, and transparency, and boosting awareness of what happens to the dogs during and between races, as a large proportion of respondents reported not knowing about specific events associated with poor management, incidence of injury, death, or the welfare of the greyhounds. In the wake of the New Zealand government placing the industry ‘on-notice’, the industry and government could respond to the loss of social license by demonstrating transparent and meaningful improvements to animal welfare in consultation with animal welfare experts. However, as greyhound racing continues to decline worldwide, calls are likely to continue for a phase-out of commercial greyhound racing.

## Figures and Tables

**Table 1:** Demographic information including gender, ethnicity, age, household income, pet ownership and if the respondent had recently donated to a pet center.

Question	*N*	Percentage
Gender		
Male	628	47.3
Female	699	52.7
Ethnicity		
Māori	104	7.8
NZ European	1025	77.2
Pacific	33	2.5
Chinese	48	3.6
Indian	25	1.9
Middle Eastern	5	0.4
Latin American	7	0.5
African	7	0.5
Other Asian	47	3.5
Other European	76	5.7
Other	67	5.
Prefer not to say	17	1.3
Age		
Under 25 years	104	7.8
25–34 years	293	22.1
35–44 years	234	17.6
45–54 years	201	15.1
55–64 years	196	14.8
65 years+	299	22.5
Household Income		
49 k and under	252	19.0
50 k to 99 k	307	23.0
100 k and over	467	35.2
Prefer not to say	301	22.7
Pet Ownership		
Yes	561	42.3
No	766	57.7
Had the respondent made a recent donation to a Pet Centre		
Yes	730	55.3
No	593	44.7

**Table 2:** Engagement with the commercial greyhound racing, betting behavior, and likelihood of attendance or gambling on greyhound racing.

Question	*N*	Percentage
Engagement with industry		
None of the above	669	50.4
Listened to or read about commercial greyhound racing in the media, news	310	23.4
Watched a greyhound race on TV	303	22.9
Talked to family or friends about commercial greyhound racing	217	16.3
Bet on a greyhound race	132	10.0
Been to a greyhound race in New Zealand	103	7.8
Signed a petition against commercial greyhound racing	97	7.3
Volunteered or donated to a greyhound rescue group	31	2.4
Written to government asking for a ban on commercial greyhound racing	19	1.4
Adopted a greyhound	12	0.9
Involved in an organization that races greyhounds	8	0.6
Owned or trained greyhounds for racing purposes	6	0.4
Been involved in an industry rehoming organization	5	0.3

**Table 3:** Knowledge of commercial greyhound racing and awareness of ‘on notice’ status.

Question	*N*	Percentage
Knowledge of greyhound racing		
I didn’t know there was commercial greyhound racing in New Zealand	167	12.6
I have heard about commercial greyhound racing in New Zealand but don’t know anything about it	725	54.6
I know a little bit about commercial greyhound racing in New Zealand	398	30.0
I know a lot about commercial greyhound racing in New Zealand	37	2.8
Awareness of ‘on notice’ status		
I am aware commercial greyhound racing is on notice	253	19.1
I was not aware commercial greyhound racing is on notice	1074	80.9

**Table 4:** Information including support, voting potential, and acceptability of racing and associated brands that support greyhound racing.

Question	*N*	Percentage
Support a ban on greyhound racing		
Yes, I would support a ban	798	60.2
No, I would not support a ban	109	8.2
I do not know enough about commercial greyhound racing to have an opinion	419	31.6
Hypothetically would sign a petition to ban		
Yes, I would sign a petition	670	50.5
No, I would not sign a petition	210	15.8
Not sure	447	33.7
Potential referendum vote		
Yes, ban commercial greyhound racing	987	74.4
No, do not ban commercial greyhound racing	340	25.6
Expressed disappointment if the government did not ban		
Strongly disagree	80	6.0
Somewhat disagree	138	10.4
Neither agree nor disagree	329	24.8
Somewhat agree	312	23.5
Strongly agree	468	35.3
Banning is ‘the right thing to do’		
Strongly agree	613	46.2
Somewhat agree	293	22.1
Neither agree nor disagree	275	20.7
Somewhat disagree	109	8.2
Strongly disagree	37	2.8
Banning would improve reputation in animal welfare		
Strongly disagree	61	4.6
Somewhat disagree	98	7.4
Neither agree nor disagree	299	22.5
Somewhat agree	438	33.0
Strongly agree	431	32.5
Opposition to greyhound racing		
Strongly support		1.3
Somewhat support		4.8
Neither support nor oppose	476	35.9
Somewhat oppose	328	24.7
Strongly oppose	429	32.3
Acceptability of brand support		
It’s fine	219	16.5
I don’t have an opinion	451	34.0
I don’t think it’s ok	657	49.5
Attitude to brands that support greyhound racing		
I would have a much more positive opinion of them	11	0.8
I would have a slightly more positive opinion	45	3.4
Not sure	495	37.3
I would have a slightly more negative opinion	312	23.5
I would have a much more negative opinion of them	464	35.0

## Data Availability

The following are available online at Cameron, K. E., Vaughan, A., McAninch, M., Briden, K., & Dale, A. Cross-sectional survey of public perception of Commercial Greyhound Racing in New Zealand. 2023. Retrieved from osf.io/m2whb on 6 July 2023.

## Supplementary Materials

The following supporting information can be downloaded at: https://www.mdpi.com/article/10.3390/ani14020207/s1, Commercial Greyhound Racing sample quota table & Commercial Greyhound Racing Questionnaire.
