# Supplementary material for: Cross-Sectional Survey of Public Perception of Commercial Greyhound Racing in New Zealand
Source: Animals (Basel). 2024 Jan 8;14(2):207. doi: 10.3390/ani14020207 (PMC10812617; doi:10.3390/ani14020207)
Supplement: Supplementary file 1 [file animals-14-00207-s001.zip › animals-2742891-supplementary.pdf]

Commercial Greyhound Racing SAMPLE QUOTA TABLE

	SampleGroup	Region	AgeGroup	Gender	TotalPopProp NZ Census	Target sample	Final sample	Final sample prop	Weight Factor
0	Auckland15-34 yearsFemale	Auckland	15-34 years	Female	6.30%	84	91	6.86%	0.918565
1	Auckland15-34 yearsMale	Auckland	15-34 years	Male	6.40%	85	69	5.20%	1.230113
2	Canterbury15-34 yearsFemale	Canterbury	15-34 years	Female	2.06%	27	28	2.11%	0.976536
3	Canterbury15-34 yearsMale	Canterbury	15-34 years	Male	2.24%	30	30	2.26%	0.992125
4	RONI15-34 yearsFemale	RONI	15-34 years	Female	4.80%	64	53	3.99%	1.202812
5	RONI15-34 yearsMale	RONI	15-34 years	Male	4.91%	65	44	3.32%	1.479465
6	ROSI15-34 yearsFemale	ROSI	15-34 years	Female	1.68%	22	25	1.88%	0.891668
7	ROSI15-34 yearsMale	ROSI	15-34 years	Male	1.71%	23	12	0.90%	1.888481
8	Wellington15-34 yearsFemale	Wellington	15-34 years	Female	1.93%	26	25	1.88%	1.026454
9	Wellington15-34 yearsMale	Wellington	15-34 years	Male	1.89%	25	20	1.51%	1.250699
10	Auckland35-44 yearsFemale	Auckland	35-44 years	Female	2.89%	38	42	3.17%	0.912849
11	Auckland35-44 yearsMale	Auckland	35-44 years	Male	2.74%	36	40	3.01%	0.909333
12	Canterbury35-44 yearsFemale	Canterbury	35-44 years	Female	1.00%	13	14	1.06%	0.950328
13	Canterbury35-44 yearsMale	Canterbury	35-44 years	Male	0.99%	13	18	1.36%	0.726844
14	RONI35-44 yearsFemale	RONI	35-44 years	Female	2.36%	31	33	2.49%	0.950814
15	RONI35-44 yearsMale	RONI	35-44 years	Male	2.20%	29	37	2.79%	0.789465
16	ROSI35-44 yearsFemale	ROSI	35-44 years	Female	0.80%	11	11	0.83%	0.967300
17	ROSI35-44 yearsMale	ROSI	35-44 years	Male	0.77%	10	10	0.75%	1.018483
18	Wellington35-44 yearsFemale	Wellington	35-44 years	Female	0.92%	12	14	1.06%	0.867488
19	Wellington35-44 yearsMale	Wellington	35-44 years	Male	0.87%	12	15	1.13%	0.768396
20	Auckland45-54 yearsFemale	Auckland	45-54 years	Female	2.84%	38	30	2.26%	1.258273
21	Auckland45-54 yearsMale	Auckland	45-54 years	Male	2.69%	36	36	2.71%	0.993325
22	Canterbury45-54 yearsFemale	Canterbury	45-54 years	Female	1.11%	15	15	1.13%	0.986080
23	Canterbury45-54 yearsMale	Canterbury	45-54 years	Male	1.08%	14	15	1.13%	0.954801
24	RONI45-54 yearsFemale	RONI	45-54 years	Female	2.73%	36	28	2.11%	1.293023
25	RONI45-54 yearsMale	RONI	45-54 years	Male	2.54%	34	23	1.73%	1.464923
26	ROSI45-54 yearsFemale	ROSI	45-54 years	Female	0.94%	12	11	0.83%	1.132734
27	ROSI45-54 yearsMale	ROSI	45-54 years	Male	0.89%	12	14	1.06%	0.841883
28	Wellington45-54 yearsFemale	Wellington	45-54 years	Female	0.96%	13	16	1.21%	0.795360
29	Wellington45-54 yearsMale	Wellington	45-54 years	Male	0.90%	12	13	0.98%	0.916132
30	Auckland55-64 yearsFemale	Auckland	55-64 years	Female	2.28%	30	26	1.96%	1.161426
31	Auckland55-64 yearsMale	Auckland	55-64 years	Male	2.16%	29	23	1.73%	1.248052
32	Canterbury55-64 yearsFemale	Canterbury	55-64 years	Female	0.99%	13	19	1.43%	0.693305
33	Canterbury55-64 yearsMale	Canterbury	55-64 years	Male	0.97%	13	13	0.98%	0.990989
34	RONI55-64 yearsFemale	RONI	55-64 years	Female	2.67%	35	40	3.01%	0.885769
35	RONI55-64 yearsMale	RONI	55-64 years	Male	2.48%	33	27	2.03%	1.219977
36	ROSI55-64 yearsFemale	ROSI	55-64 years	Female	0.91%	12	11	0.83%	1.102063
37	ROSI55-64 yearsMale	ROSI	55-64 years	Male	0.90%	12	12	0.90%	0.993003
38	Wellington55-64 yearsFemale	Wellington	55-64 years	Female	0.80%	11	13	0.98%	0.820918
39	Wellington55-64 yearsMale	Wellington	55-64 years	Male	0.75%	10	12	0.90%	0.833887
40	Auckland65+ yearsFemale	Auckland	65+ years	Female	2.70%	36	31	2.34%	1.155376
41	Auckland65+ yearsMale	Auckland	65+ years	Male	2.31%	31	40	3.01%	0.766709
42	Canterbury65+ yearsFemale	Canterbury	65+ years	Female	1.37%	18	22	1.66%	0.827362
43	Canterbury65+ yearsMale	Canterbury	65+ years	Male	1.17%	16	16	1.21%	0.970643
44	RONI65+ yearsFemale	RONI	65+ years	Female	3.75%	50	52	3.92%	0.955872
45	RONI65+ yearsMale	RONI	65+ years	Male	3.31%	44	58	4.37%	0.757118
46	ROSI65+ yearsFemale	ROSI	65+ years	Female	1.27%	17	26	1.96%	0.646102
47	ROSI65+ yearsMale	ROSI	65+ years	Male	1.15%	15	17	1.28%	0.896057
48	Wellington65+ yearsFemale	Wellington	65+ years	Female	1.04%	14	23	1.73%	0.600143
49	Wellington65+ yearsMale	Wellington	65+ years	Male	0.88%	12	14	1.06%	0.832243
TOTAL					100.00%	1327	1327	100%	

Commercial Greyhound Racing QUESTIONNAIRE

Section A: Current Behaviour

Intro/Landing Page
Thanks for clicking through to take part in this survey. Today we're interested in your opinions on commercial greyhound racing in New Zealand.

Familiarity
How familiar are you with commercial greyhound racing in New Zealand?
[single response]

I didn't know there was commercial greyhound racing in New Zealand
I have heard about commercial greyhound racing in New Zealand but don't know anything about it
I know a little bit about commercial greyhound racing in New Zealand
I know a lot about commercial greyhound racing in New Zealand

Involvement
Have you <u>ever</u> done any of the following:
Please select all that apply.
[multi select, randomize]

Been to a greyhound race in New Zealand
Watched a greyhound race on TV
Bet on a greyhound race
Adopted a greyhound
Volunteered or donated to a greyhound rescue group
Listened to or read about commercial greyhound racing in the media, news
Talked to family or friends about commercial greyhound racing
Involved in an organisation that races greyhounds
Owned or trained greyhounds for racing purposes
Been involved in an industry rehoming organisation
Signed a petition against commercial greyhound racing
Written to government asking for a ban on commercial greyhound racing
None of the above [anchor, exclusive]

Gambling question
In the last year have you bet on any of the following:
Please select all that apply.
[multi select, randomize]

Greyhound racing
Horse racing (harness and/or thoroughbred)
Rugby
Cricket
Golf
Tennis
Football / soccer
Basketball

Baseball
Ice hockey
American football / NFL
Other sports event
Have not bet on any of these lock, exclusive

Section 2: Perceptions of Greyhound Racing

Support
Do you support the continuation of commercial greyhound racing in New Zealand?
[single select]

Strongly support commercial greyhound racing
Somewhat support commercial greyhound racing
Neither support nor oppose commercial greyhound racing
Somewhat oppose commercial greyhound racing
Strongly oppose commercial greyhound racing

Interest-Track
How likely are you to attend a live commercial greyhound race in the next 6 months?
[single select]

Very unlikely
Quite unlikely
Not sure
Quite likely
Very likely

Interest-Track2
How likely are you to bet / gamble on commercial greyhound racing in the next 6 months?
[single select]

Very unlikely
Quite unlikely
Not sure
Quite likely
Very likely

Positive-Negative
Some people think there are positives to commercial greyhound racing while others think there are negatives. On balance, do you think there are more positive or negative aspects?
[single select]

Mainly positive
Slightly more positive
Probably balanced

Slightly more negative
Mainly negative

Pros

What would you say, if any, are the positive impacts of commercial greyhound racing in New Zealand?
[multiselect, randomize]

Good entertainment
Contributes to the New Zealand economy
Greyhounds enjoy racing
Preserves the greyhound breed
Supports legal gambling
Promotes animal education (teaching animal husbandry)
Promotes cultural values (traditions)
Creates jobs, financial opportunity
Contributes to general human well-being (relaxation, social opportunities)
Brings the community together
Another impact (please specify): [anchor]
There are no positive impacts of commercial greyhound racing [anchor, exclusive]

Cons

And what would you say, if any are negative impacts of commercial greyhound racing in New Zealand?
[multiselect, randomize]

Endangers greyhound health and welfare at the race track
Endangers greyhound health and welfare off the race track
Greyhounds being euthanised
Contributes to dog overpopulation
Promotes gambling
Creates a poor international reputation for New Zealand
Normalises exploitation of animals for entertainment
Greyhounds can be difficult to rehome due to early life experiences
Contributes to the use of illegal or banned substances (e.g. greyhounds testing positive for drugs)
Another impact (please specify): [anchor]
There are no negative impacts of commercial greyhound racing [anchor, exclusive]

Good-Life

Do you think greyhounds that are bred for racing have a good life?
[single select]

Yes
No
Not sure

GRNZ-Aware
Have you heard of Greyhound Racing New Zealand (GRNZ)?
[single select]

Yes
No
Not sure

GRNZ-Welfare
Greyhound Racing New Zealand is the governing body for commercial greyhound racing in New Zealand and provides governance, support and assistance to the affiliated clubs in the sport of greyhound racing. GRNZ is responsible for all the administration of all supporting functions such as racing and breeding support, animal welfare, promotion of greyhound racing, to race stakes and schedules.

Do you believe GRNZ is fulfilling its role in animal welfare?
[single select]

Yes
No
I don't know enough to have an opinion

Government
New Zealand is one of only seven countries in the world where commercial greyhound racing is legal. The Government has two Ministers who are responsible for oversight of the welfare of greyhounds in the commercial greyhound industry: The Minister for Racing, Hon. Kieran McAnulty, and the Associate Minister of Agriculture (Animal Welfare), Hon. Meka Whaitiri.

Do you believe the government does enough to keep greyhounds involved in the commercial racing industry safe from harm?
[single select]

Yes, I believe the government does enough
No, I do not believe the government does enough
I don't believe the government should be involved in commercial greyhound racing oversight
I don't know enough to have an opinion

Betting-GH
Do you think betting on commercial greyhound racing is an acceptable form of entertainment?
[single select]

Yes
No
Not Sure

Gambling2

In general, do you think any type of gambling is acceptable?
[single select]

Yes
No
Not Sure

Section 3: Deep dive into specific areas

Welfare
Below are some facts about greyhound health and welfare in the NZ commercial greyhound industry. To what extent do you feel that they are acceptable part of the industry vs. something we should work to remedy?
[single choice grid]
]rows]

- There are no restrictions on how many greyhounds can be housed on a single property
- Some kennels have more than 100 dogs which makes it difficult to socialise puppies
- More greyhound puppies were bred for the industry in the 2020/2021 season than in the previous 4 seasons
- The majority of racing greyhounds are bred via surgical artificial insemination
- A number of greyhounds have been reported to arrive at rehoming facilities blind, possibly due to high worm burdens
- Many young greyhounds that go to a rehoming centre have serious dental issues, many requiring extractions
- This year kennel audits issued between 200-300 improvement notices for issues including unvaccinated dogs, unapproved medications being used, treatment records not being kept, an increase in dental issues, more dogs found at some kennels than official records show
- Only approximately 7% of racing greyhounds are tested for banned substances at races (tests per race starts)
- There is no requirement for greyhound trainers to demonstrate they have read or understood industry standards related to welfare
- Trainers who have repeatedly been found to have failed to provide proper care for the welfare of their dogs continue to be part of the industry

[columns]
An acceptable part of racing
Something that needs to be improved
Something that is completely unacceptable
Not sure / Undecided

Euthanasia and injuries
Below are some facts about greyhound injuries and euthanasia. To what extent do you feel that they are acceptable part of the industry vs. something we should work to remedy?
[single choice grid]
[rows]

- 80% of on track injuries occur on the first bend but straight tracks have not been introduced in New Zealand

- Vets at the track have less than 1 minute to perform a physical exam to assess if greyhounds are fit to race
- There were 206 injuries on the track in the 2021-2022 racing season
- 83 dogs suffered a fracture on the track in the 2021-2022 racing season
- 9 dogs were euthanised at the track due to catastrophic injuries in the 2021-2022 racing season
- While euthanasia rates have decreased, serious injury rates have actually increased in recent seasons
- Off track deaths and euthanasia are included in annual reports but “off track” health or injury data is not reported or recorded in an industry database

[columns]

An acceptable part of racing
Something that needs to be improved
Something that is completely unacceptable
Not sure / Undecided

Rehoming
There are many charities and organisations that help with rehoming greyhounds, however there remain issues in this area.

Below are some facts about rehoming, to what extent do you feel that they are acceptable part of the industry vs. something we should work to remedy?

[single choice grid]
[rows]

- More greyhounds are bred and imported than are needed for racing
- GRNZ has expanded its rehoming programme but has not reduced the number of puppies they are breeding, established any form of public reduction targets, population projections, or estimated the number of dogs needed for the industry each year
- Some greyhounds are not adequately socialised as puppies and can find normal household noises and experiences, meeting new people or animals or any novel experience frightening and distressing
- Some greyhounds miss out on vital training as puppies and therefore may struggle to adapt to life in a home environment
- Some greyhounds, commonly referred to as “spooks”, show a severe fear response to any new experience or person
- Common behavioural issues reported in ex-racing greyhounds include predatory aggression, fear, and separation anxiety
- The greyhound racing industry and dog rescues across New Zealand are currently finding it harder to find homes for dogs, resulting in dogs waiting longer in rehoming kennels

[columns]

An acceptable part of racing
Something that needs to be improved
Something that is completely unacceptable
Not sure / Undecided

Change

An independent review of commercial greyhound racing was conducted and the recommendations are provided in the 2021 Robertson Review. Below is a list of the recommendations. Which of the recommendations below do you personally think are important to implement?

Please select all recommendations you feel should be implemented.
[multiselect, randomize]

- Data available on the position of all animals from birth to death (*improves transparency and data reporting which were both identified as issue in this review of the industry*)
- An assessment of the number of animals required and the avoidance of over breeding (*to reduce overpopulation and pressure on rehoming facilities*)
- A rationalisation of all relevant rules, policies, and standards which covers all aspects of dog welfare throughout the industry (*to make animal welfare requirements clear for industry participants*)
- Socialisation programmes for all greyhounds so they can have the potential to be meaningfully rehomed later in life (*reduces risk of behaviour problems such as predatory aggression, and severe fear responses*)
- Thorough professional kennel visits (to check for compliance with welfare standards)
- Continuous surveillance of tracks including a serious assessment of the introduction of straight tracks (*straight tracks could reduce track day injuries*)
- A rigorous assessment of animal welfare in large scale operations (*oversight of animal welfare at kennels with large number of dog*s)
- A reintroduction of a standalone Animal Welfare Manager whether within GRNZ or in the RIB (*animal welfare expertise to advise industry*)
- A Health and Welfare Committee with full participation of all relevant stakeholders and the ability to implement and effect change (*to ensure oversight of industry and animal welfare expertise*)
- A continuation, as promised, of reporting to both the relevant Ministers and the Racing Integrity Board (RIB) on operations and progress (*previous reporting was stopped by the industry despite concerns raised by the health and welfare committee and government requests that they continue*)
- All of the above are important to implement [exclusive, anchor]
- None of the above are important to implement [exclusive, anchor]

On-Notice

Are you aware that commercial greyhound racing is "on notice" meaning that it could be shut down if greyhound welfare, transparency and data recording is not addressed?

I am aware commercial greyhound racing is on notice
I was not aware commercial greyhound racing is on notice

Section 4: Driving change/ PR questions

Ban
Would you support a ban on commercial greyhound racing in New Zealand?
[single choice]

Yes, I would support a ban
No, I would not support a ban
I do not know enough about commercial greyhound racing to have an opinion

Petition
Hypothetically, would you be willing to sign a petition supporting a ban on commercial greyhound racing?
[single choice]

Yes, I would sign a petition
No, I would not sign a petition
Not sure

Referendum
If a referendum was held tomorrow to ban commercial greyhound racing how would you vote?
[single choice]

Yes, ban commercial greyhound racing
No, do not ban commercial greyhound racing

Right-Thing
Do you disagree or agree with the following statement?

Banning commercial greyhound racing in New Zealand is the right thing to do.
[single choice]

Strongly agree
Somewhat agree
Neither agree nor disagree
Somewhat disagree
Strongly disagree

Repu-Impact
New Zealand is only one of 7 countries in the world that still allows commercial greyhound racing.
Do you agree or disagree with the following statements?

[rows, randomize]
Banning commercial greyhound racing would improve New Zealand's reputation for animal welfare
I would be disappointed in the government if they did not ban commercial greyhound racing when given the opportunity

[columns]

Strongly agree
Somewhat agree
Neither agree nor disagree
Somewhat disagree
Strongly disagree

Sponsorship
Do you think it's okay for brands to sponsor and financially support commercial greyhound racing?
[single choice]

It's fine
I don't have an opinion
I don't think it's ok

Sponsorship2
What do you think of brands that do sponsor and financially support commercial greyhound racing?
[single choice]

I would have a much more positive opinion of them
I would have a slightly more positive opinion
Not sure
I would have a slightly more negative opinion
I would have a much more negative opinion of them

[do not ask if Ban = No, I would not support a ban]
Reason-Ban-OE
Given everything you have seen is this survey, what do you think is the most compelling reason to ban commercial greyhound racing?

[open end]

Section 5: Demos

Volunteer

Just a few final questions for classification purposes.

Have you ever volunteered for or made a financial donation to an animal support or rescue group?
[single choice]

Yes
No

Urban-Rural
Do you live in a rural or urban part of New Zealand?
[single choice]

Rural
Urban

Thank and close survey.
